# Four new species of Gesneriaceae from Yunnan, Southwest China

**DOI:** 10.3897/phytokeys.130.34001

**Published:** 2019-08-29

**Authors:** Bin Yang, Hong-Bo Ding, Kai-Cong Fu, Yi-Kai Yuan, Han-Yu Yang, Jian-Wu Li, Li-Xia Zhang, Yun-Hong Tan

**Affiliations:** 1 Southeast Asia Biodiversity Research Institute & Center for Integrative Conservation, Xishuangbanna Tropical Botanical Garden, Chinese Academy of Sciences, Menglun, Mengla, Yunnan 666303, China Xishuangbanna Tropical Botanical Garden, Chinese Academy of Sciences Mengla China; 2 Key Laboratory of Dai and Southern medicine of Xishuangbanna Dai Autonomous Prefecture, Yunnan Branch, Institute of Medicinal Plant,Chinese Academy of Medical Sciences, Jinghong, Yunnan 666100, China Pu’er Traditional Ethnomedicine Institute Pu’er China; 3 Pu’er Traditional Ethnomedicine Institute, Simao, Pu’er, Yunnan 665000, China Institute of Medicinal Plant,Chinese Academy of Medical Sciences Jinghong China

**Keywords:** China, Gesneriaceae, taxonomy, *
Petrocosmea
*, *
Didymocarpus
*, *
Henckelia
*

## Abstract

Four new species of Gesneriaceae from Yunnan, southwest China, are described and illustrated. They are *Petrocosmea rhombifolia*, *Petrocosmea tsaii*, *Didymocarpus brevipedunculatus*, and *Henckelia xinpingensis*. Diagnostic characters between the new species and their morphologically close relatives are provided. Their distribution, ecology, phenology, and conservation status are also described.

## Introduction

Gesneriaceae (Lamiales) consists of ca. 150 genera and around 3500 species of perennial herbs, shrubs or small trees, with the main distribution in the tropics and subtropics (Weber et al. 2013; Möller et al. 2016a; Middleton et al. 2018). In China, there are >600 species in 44 genera (Möller et al. 2016a, b; Xu et al. 2017). Major taxonomic changes have been implemented in accordance with phylogenetic evidence affecting the classification of Chinese Gesneriaceae, so many morphologically defined genera have been split or merged, or new genera described (reviewed in Möller et al. 2016a). Southern China harbours most species of Gesneriaceae, and Guangxi, Yunnan, Guizhou and Guangdong are species richness regions in Gesneriaceae (Xu et al. 2017).

During botanical surveys from 2012 to 2018 in Yunnan, several specimens of Gesneriaceae were collected. From the vegetative forms and flower characters, they were identified as members of *Petrocosmea* Oliv. (Oliver 1887), *Didymocarpus* Wall. (Wallich 1819), and *Henckelia* Spreng. (Sprengel 1817), respectively. *Petrocosmea* has more than 50 known species distributed in China, Vietnam, Thailand and India (Han et al. 2018b); *Didymocarpus* has approximately 70 species range from northwest India, eastwards through Nepal, Bhutan, northeast India, Myanmar, to southern China, Vietnam, Laos, Cambodia, Thailand, the Malay Peninsula and northwards to Sumatra (Weber and Burtt 1998; Weber et al. 2000; Möller et al. 2016a; Hong et al. 2018); *Henckelia* has 64 known species found in Sri Lanka, southern and north-eastern India, Nepal, Bhutan, southern China, northern Laos, northern Vietnam and northern Thailand (Weber et al. 2011; Sirimongkol et al. 2019). After thorough comparisons of diagnostic morphological, anatomical features and herbarium specimens available at BM, E, HITBC, K, KUN, NYBG and P with similar taxa of *Petrocosmea*, *Didymocarpus*, and *Henckelia*, and consulting the relevant literature for *Petrocosmea* (Wang 1985; Wang et al. 1990, 1998; Burtt 1998a; Li and Wang 2004; Wei and Wen 2009; Gou et al. 2010; Middleton and Triboun 2010; Zhao and Shui 2010; Shaw 2011; Xu et al. 2011; Qiu and Liu 2015; Qiu et al. 2011, 2012, 2015a, 2015b; Wang et al. 2013; Zhang et al. 2013; Han et al. 2017, 2018a, 2018b), *Didymocarpus* (Wang et al. 1998; Burtt 1998b, 1999, 2001; Weber et al. 2000; Hilliard 2001; Li and Wang 2004; Nangngam and Maxwell 2013; Wen et al. 2013; Li and Li 2014; Nangngam and Middleton 2014; Phuong et al. 2014; Li and Wang 2015; Cai et al. 2016; Joe et al. 2016; Hong et al. 2018), and *Henckelia* (Wang et al. 1998; Weber and Burtt 1998; Burtt 2001; Weber et al. 2011; Middleton et al. 2010; Ranasinghe et al. 2016; Sirimongkol et al. 2019) from China and adjacent regions, it was confirmed that the four species were new to science. Here, they are described and illustrated with photographs and drawings.

## Material and methods

Morphological observations were carried out on living plants in the field and greenhouse, as well as dried specimens. All morphological characters were measured under a dissecting microscope and descriptions were made following the terminology presented by Wang 1985 and Wang et al. 1998. Literature studies included all relevant monographs of *Petrocosmea*, *Didymocarpus*, and *Henckelia*, and recently published papers (see introduction), and also similar taxa, i.e. *Petrocosmea rosettifolia* C. Y. Wu ex H. W. Li (Li 1983, Wang et al. 1998, Zhao and Shui 2010), P. kerrii Craib var. kerrii (Craib 1918, Wang 1985, Wang et al. 1998), *P. menglianensis* H. W. Li (Li 1983, Wang 1985, Wang et al. 1998), *Didymocarpus purpureobracteatus* W.W. Smith (Smith 1912, Wang et al. 1998), and *Henckelia pumila* (D. Don) A. Dietr. (Dietrich 1831, Wang et al. 1998, Weber et al. 2011). Specimens at BM, E, HITBC, K, KUN, NYBG, P, and PE were checked and the images of type specimens were also obtained from the Chinese Virtual Herbarium (CVH, http://www.cvh.ac.cn), KUN (http://kun.kingdonia.org) and JSTOR Global Plants (http://plants.jstor.org/). Species Conservation Assessment was undertaken using the IUCN methodology (IUCN 2012; IUCN Standards and Petitions Subcommittee 2016).

## Taxonomic treatments

### 1. Petrocosmea rhombifolia

Taxon classificationPlantaeLamialesGesneriaceae

Y.H.Tan & H.B.Ding sp. nov.

BA1A187FEECC51E49F29B50FFD9752A8

urn:lsid:ipni.org:names:60479351-2

Figures 1 , 2

#### Diagnosis.

*Petrocosmea rhombifolia* is similar to *P. rosettifolia*, but differs from the latter in having rhombic leaf blades (vs. broadly ovate to orbicular or broadly elliptic) and much longer petiole to 15 cm long (vs. to 4 cm long); the flowers have upper white lip (vs. purple-blue flowers throughout), corolla adaxial lip 14–15 × 9–10 mm (vs. ca. 5 mm), abaxial lip 27–28 × 12–14 mm (vs. ca. 7–8 × 6–8 mm), and flowering March-April (vs. October).

#### Type.

CHINA. Yunnan Province: Lancang County, Laba village, 22°36'42.52"N, 99°42'57.10"E, a.s.l. 1900 m, 1 April 2017, Y.H. Tan & H.B. Ding, *T0119* (holotype: HITBC!).

**Figure 1.**
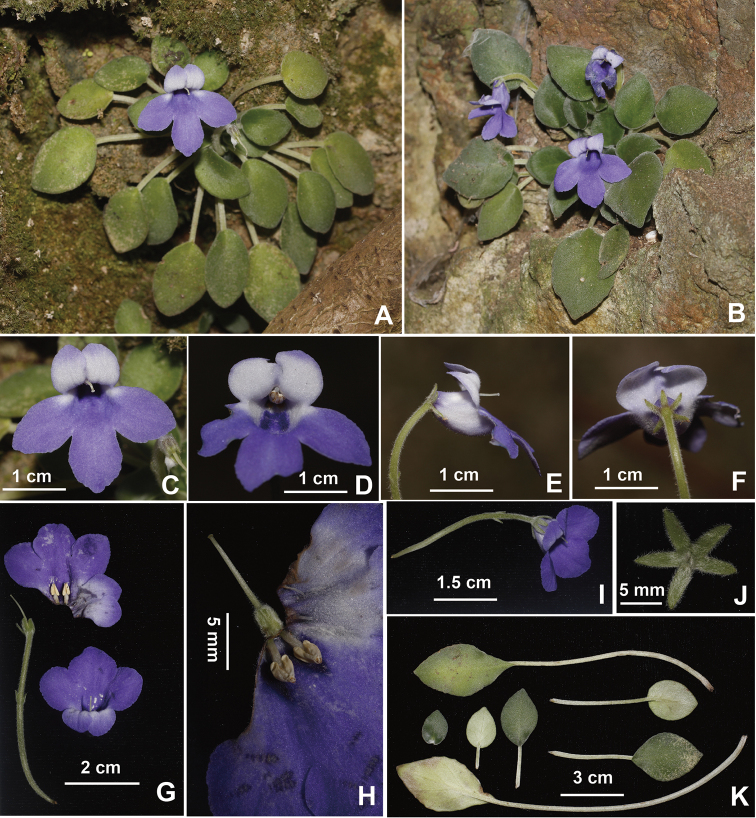
*Petrocosmea rhombifolia* Y.H.Tan & H.B.Ding, sp. nov. **A, B** Habit **C, D** flower in front view **E** flower in side view **F** flower in back view **G** flower **H** dissected corolla (showing pistil and stamens) **I** cyme **J** calyx in abaxial view **K** leaves. Photographed by H.B. Ding.

**Figure 2.**
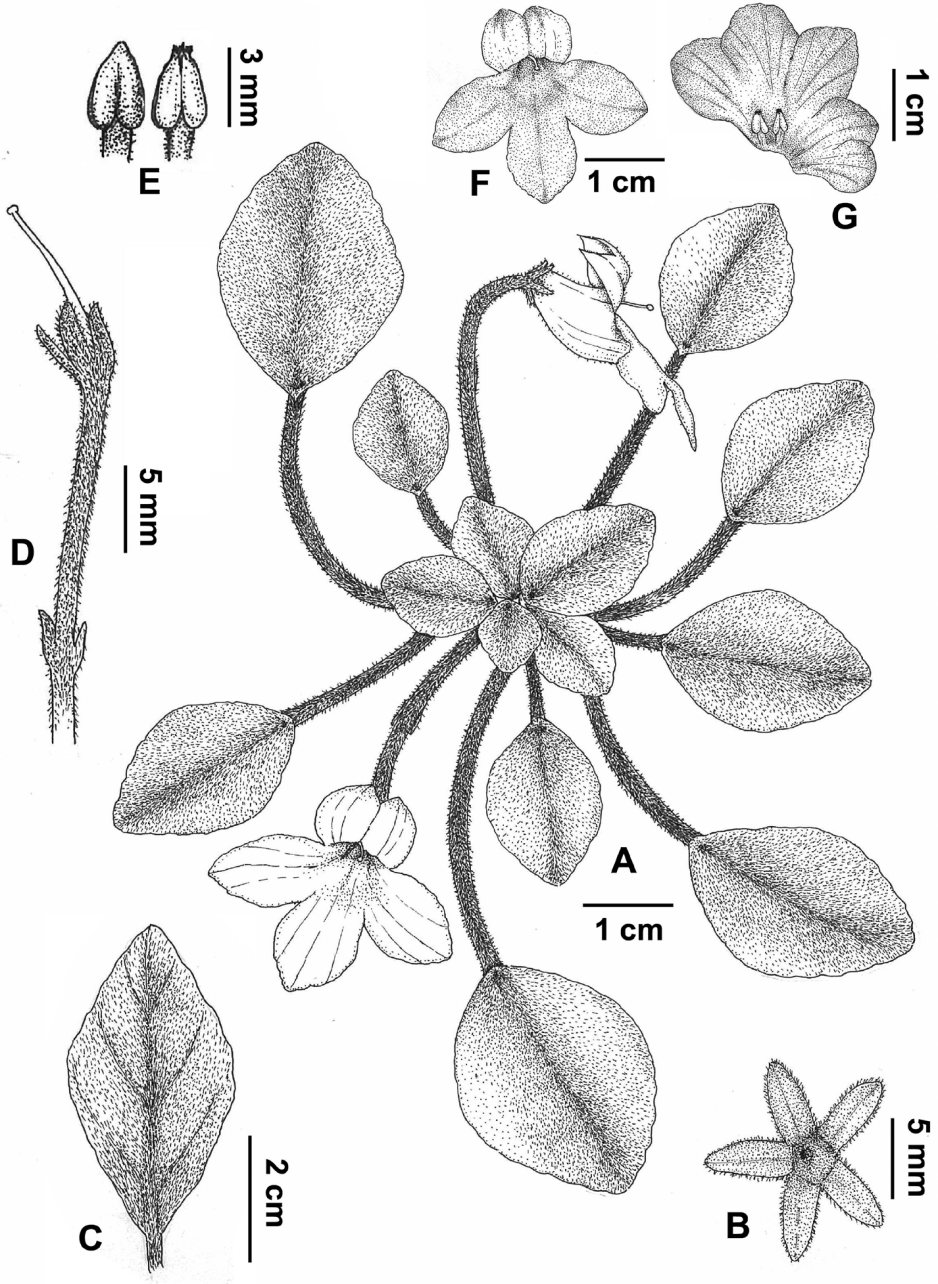
*Petrocosmea rhombifolia* Y.H.Tan & H.B.Ding, sp. nov. **A** Habit **B** calyx in adaxial view **C** leaf blade in abaxial view **D** pedicel with calyx and pistil **E** anthers **F** corolla in front view **G** dissected corolla. Drawn by Zhen-Meng Yang.

#### Description.

Perennial herb with short rhizomatous stem and crowded fibrous roots. **Leaves** 14 to 25, all in basal rosette; petioles 0.5–15 cm long, densely white pubescent to sericeous; leaf blades ovate or ovate to rhombic, 1.5–5.3 × 1.3–2.8 cm, rounded or cuneate at base, with nearly entire or slightly repand margins and acute or obtuse apex, densely pubescent to sericeous on both surfaces; lateral veins abaxially conspicuous, 2–3 on each side. **Inflorescences** 1–flowered, 4–6 cm long; **Peduncles** 2.5–3.2 cm long, pedicels 1.6–2.0 cm, densely pubescent to sericeous; **Bracts** 2, opposite, subulate, 2–3 mm. **Calyx** actinomorphic, equally divided into 5 lobes from base, lobes lanceolate, 4–5 mm, sparsely pubescent inside, densely sericeous outside. **Corolla** light blue, sparsely pubescent to puberulous outside, sparsely puberulent or subglabrous inside; tube 5–6 mm, sometimes with 2 ovate brown spots inside below the stamens; throat light blue or whitish blue with 2 oblong deep blue blotches; adaxial lip ca. 14–15 × 9–10 mm, semi-orbicular, light blue or whitish blue, distinctly 2-lobed, lobes reflexed, with rounded apex and repand margin; abaxial lip ca. 27–28 × 12–14 mm, blue, 3-lobed to or over the middle, with sub-orbicular to obovate lobes, lobes with rounded apex and repand to slightly crenate margin. **Stamens** 2, about 6 mm long, adnate to the base of the corolla tube; filaments about 3 mm long, sparsely pubescent; anthers ovate, about 3 mm long, dehiscence poricidal, glabrous, dorsifixed, coherent at apex. **Staminodes** 3, ca. 2 mm, adnate to the corolla tube at the base, sub-glabrous. **Pistil** ca. 1.1 cm; ovary densely villous, oblate, ca. 3 mm; style ca. 8 mm, sparsely pubescent near base; stigma capitate. **Fruit** a short capsule, 8–10 mm long.

#### Etymology.

The new species is named after its rhombic leaf blades.

#### Vernacular name.

Chinese mandarin: ling ye shi hu die (菱叶石蝴蝶).

#### Phenology.

Flowering March-May and fruiting April-June.

#### Distribution and habitat.

*Petrocosmea rhombifolia* grows on moist rock faces in limestone forest, at elevation ca. 1900 m in Laba, Lancang County.

#### Conservation status.

*Petrocosmea rhombifolia* has hitherto only been found at its type locality in Laba, Lancang County. There is very limited information about its natural distribution; a further detailed investigation of the same habitats will help to identify additional populations and individuals of this new species. The lack of sufficient data currently does not allow a risk evaluation and the species can be regarded at present as Data Deficient (DD) according to the IUCN Red List Categories and Criteria (IUCN 2012).

#### Note.

*Petrocosmea rhombifolia* has ovate leaf blades with pubescence on the surfaces that are similar to *P. rosettifolia*, but mainly different from the leaf blade and flower characters. A comparative list of diagnostic characters of the new species and *P. rosettifolia* is given in Table 1.

**Table 1.** Morphological comparison between *Petrocosmea rhombifolia* sp.nov. and *P. rosettifolia* C. Y. Wu ex H. W. Li.

Characters	*P. rhombifolium*	*P. rosettifolia*
**Leaf blade**		
Shape and size	ovate or ovate to rhombic, 1.5–5.3 × 1.3–2.8 cm	broadly ovate to orbicular or broadly elliptic, 0.5–4.0 × 0.4–3.0cm
Margin	nearly entire or slightly repand	entire to crenulate-serrulate toward apex
lateral veins	conspicuous, 2–3 pairs	inconspicuous
Base	rounded or cuneate	broadly cuneate to cuneate
Apex	acute or obtuse	broadly acute
indumentum	densely white pubescent to siliceous	densely appressed puberulent or sericeous to tomentose
Petiole	to 15 cm long	to 4 cm long
**Cymes**	1-flowered	1-flowered
**Corolla**		
Calyx	actinomorphic, equally divided into 5 lobes from base	actinomorphic, equally divided into 5 lobes from base
colour and indumentum	light blue, upper lip white, outside sparsely pubescent to puberulous, inside sparsely puberulent or subglabrous	purple-blue throughout, outside sparsely puberulent, inside glabrous
adaxial lip	14–15 × 9–10 mm, distinctly 2-lobed with the two lobes reflexed	ca. 5 mm, distinctly 2-lobed
abaxial lip	27–28 × 12–14 mm	ca. 8 × 7 mm
Throat	whitish light blue or somewhat light blue with 2 oblong deep blue blotches	white
Tube	5–6 mm	ca. 5 mm
**Stamens**		
filaments	ca. 3 mm, sparsely pubescent	ca. 3 mm, minutely hispid
anthers	ca. 3 mm	ca. 1 mm, beakless
**Pistil**	ca. 1.1 cm	ca. 1 cm
Ovary	oblate, densely villous	elliptic-ovoid, appressed puberulent
Style	ca. 8 mm, sparsely pubescent near base	5–7 mm, sparsely puberulent near base
Flowering time	March to May	October

### 2. Petrocosmea tsaii

Taxon classificationPlantaeLamialesGesneriaceae

Y.H.Tan & JianW.Li sp. nov.

FE5A4D10AA0F5A0FAD7CD554E131413E

urn:lsid:ipni.org:names:60479352-2

Figures 3 , 4

#### Diagnosis.

*Petrocosmea tsaii* is similar to P. kerrii var. kerrii and *P. menglianensis* in having elliptic leaf blade, oblique and rounded leaf base, acute leaf apex, ellipsoid anthers with brevirostrate apex; but it can be easily distinguished from the two similar taxa by its bluish purple corolla (vs. white) and much longer inflorescences. *Petrocosmea tsaii* also differs from P. kerrii var. kerrii by having actinomorphic calyx (vs. zygomorphic), and differs from *P. menglianensis* by its leaf blade abaxially densely villous (vs. pubescent along midrib and lateral veins).

#### Type.

CHINA. Yunnan Province: Mengla county, Menglun, Mengxing, 21°49'N, 101°23'E, a.s.l. 1200 m, 13 Sep. 2016, *Jian-Wu Li 4577* (holotype: HITBC!).

#### Description.

Perennial herb with short rhizomatous stem. **Leaves** 8–15, in basal rosette; inner leaves with petioles short or absent, ovate or suborbicular; outer leaves with long petioles, elliptic or ovate to widely ovate; 1.5–10.5 × 1.2–8.2 cm, apex acute to rounded, base rounded to subcordate, sometimes oblique, margin crenate, densely villous on abaxial surfaces, sparsely pubescent to puberulous on adaxial surface; **lateral veins** 4–10 on either side of midrib, adaxially impressed, abaxially conspicuous; **petioles** up to 10 cm long, densely white villous. **Inflorescences** 6.0–14.5 cm long; Peduncles 3.5–11.0 cm long, 2.0–2.5 mm in diam., densely villous and with glandular hairs; **bracts** 2–3, ovate to broadly ovate, or somewhat leaf like, with 4–5 lateral veins on side, ovate-elliptic, 8–19 × 6–18 mm; **cymes** usually 3–6(–8)-flowered, **hypopodium** 0.5–3.5 cm, **pedicels** 1.2–2.3 cm, villous and with glandular hairs; **bracteoles** 2, opposite, linear-lanceolate, 3.5–8.3 × 1.5–2 mm. **Calyx** actinomorphic, equally divided into 5 lobes from base, lobes linear-lanceolate, 6–7 × 1–1.5 mm, internally sparsely with glandular hairs, externally villous and with glandular hairs, margin with 1–3 linear teeth above middle. **Corolla** 10.5–12 mm long, externally sparsely puberulous to glabrous, internally glabrous; tube 4–4.5 mm; throat dark bluish purple; adaxial lip ca. 7–9 × 10–12 mm, indistinctly 2-lobed with the two lobes reflexed, lobes semi-orbicular, with rounded apex and entire margin, base white; abaxial lip ca. 16–20 × 9–11 mm, 3-lobed to the middle, lobes semi-orbicular, with rounded to obtuse apex, bluish purple. **Stamens** 2, 4–4.5 mm long, adnate to the base of the corolla tube; anthers adnate face to face; filaments 1.5–2 mm long, with short glandular hairs near base; anther ovoid to ellipsoid, 3–3.5 mm long, with brown capitate-glandular hairs, dorsifixed, apex brevirostrate. **Staminodes** 2–3, ca. 1 mm, adnate to the corolla tube at the base, linear, glabrous. **Pistil** 11–12 mm; ovary 3–3.5 mm long, narrowly ovoid, sparsely pubescent and with yellow glandular hairs; style 7.5–9 mm, sparsely with yellow glandular hairs at base, upper part glabrous; stigma capitate. **Fruit** a short capsule, 10–12 mm long.

**Figure 3.**
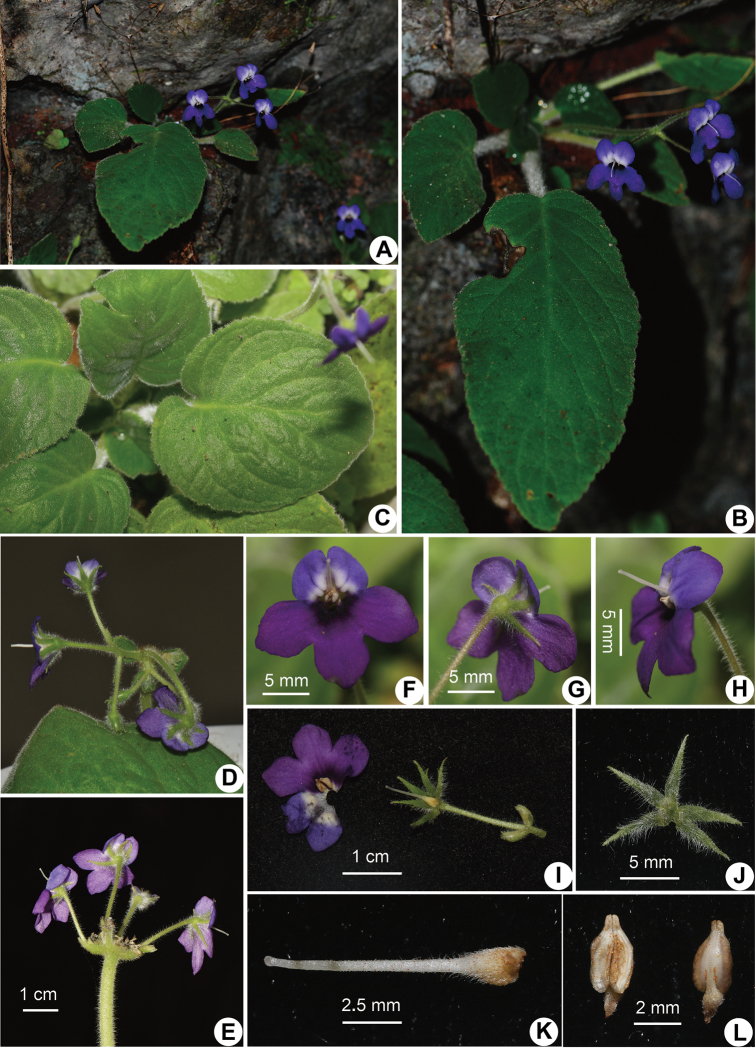
*Petrocosmea tsaii* Y.H.Tan & JianW.Li, sp. nov. **A, B** Habit **C** leaves **D, E** inflorescence **F** flower in front view **G** flower in back view **H** flower in side view **I** dissected flower **J** calyx in abaxial view **K** pistil **L** stamens. Photographed by J.W.Li, Y.H.Tan & H.B.Ding.

**Figure 4.**
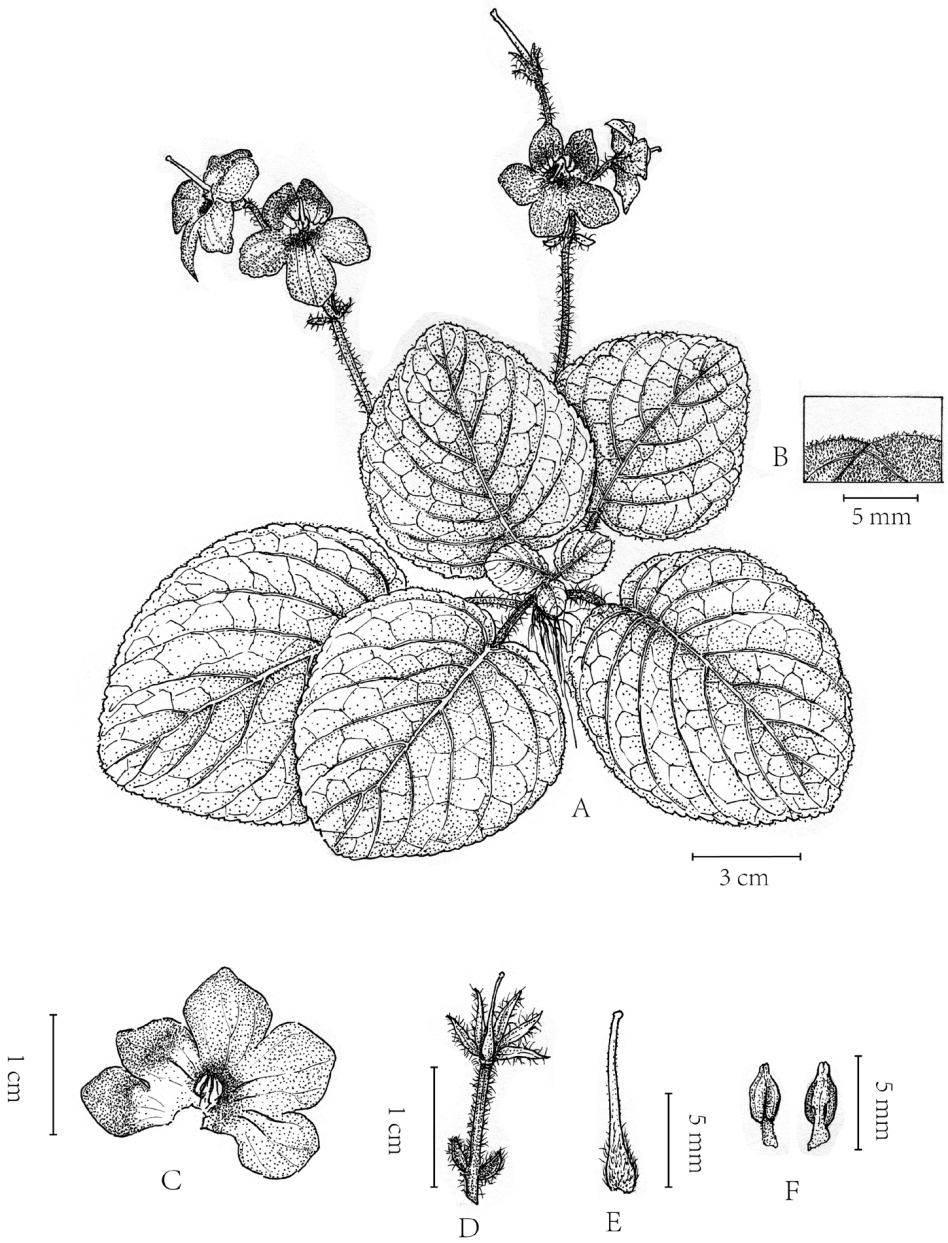
*Petrocosmea tsaii* Y.H.Tan & JianW.Li, sp. nov. **A** Habit **B** adaxial Leaf surface indumentum **C** corolla (Dissected) **D** pedicel with bracteoles, calyx and pistil **E** pistil **F** stamens. Drawn by Zhengmeng Yang.

#### Etymology.

The specific epithet commemorates the late Prof. Cai Xitao (Tsai Hse-Tao), who was the founder of Xishuangbanna Tropical Botanical Garden (XTBG) and devoted all his life to the study of Chinese plants.

#### Vernacular name.

Chinese mandarin: Cai Shi Shi Hu Die (蔡氏石蝴蝶)

#### Phenology.

Flowering September-October and fruiting October-November.

#### Distribution and habitat.

The species grows on moist rock faces in limestone forests, Mengla County, Yunnan, China.

#### Conservation status.

Due to insufficient field surveys so far, very few details about its natural distribution and population status are currently known. The lack of sufficient data does not allow a risk evaluation and the species can be regarded at present as Data Deficient (DD) according to the IUCN Red List Categories (IUCN 2012).

#### Note.

A comparison of the diagnostic characters of the new species and P. kerrii var. kerrii, *P. menglianensis* is given in Table 2.

**Table 2.** Morphological comparison among *Petrocosmea tsaii* sp. nov., Petrocosmea kerrii Craib var. kerrii and *Petrocosmea menglianensis* H. W. Li.

Characters	*P. tsaii*	P. kerrii var. kerrii	*P. menglianensis*
**Leaf blade**			
shape and size	elliptic or ovate to widely ovate; 1.5–10.5 × 1.2–8.2 cm	elliptic to rhombic-elliptic or ovate, 1.8–13.5 × 1.2–8.5 cm	elliptic to elliptic-ovate, 7.5–8.5 × 5–6 cm
margin	crenate	dentate	irregularly dentate
Base	sometimes oblique, rounded to subcordate,	usually oblique, broadly cuneate to rounded	oblique, rounded to cuneate
Apex	acute to rounded	broadly acute to obtuse, rarely rounded	broadly acute to obtuse
indumentums	adaxially sparsely pubescent to puberulous, abaxially densely villous	adaxially and abaxially densely hirsute to densely puberulent	adaxially rust-brown pubescent, abaxially rust-brown pubescent along midrib and lateral veins
**Bracts**	ovate to broadly ovate, or somewhat leaf like, 8–19 × 6–18 mm	lanceolate, ca. 2.0 × 0.5 mm	subulate to lanceolate, 3–4 × 1.0–1.5 mm
Calyx	аctinomorphic	zygomorphic	actinomorphic
Corolla colour	bluish purple	white	white
Throat of corolla	dark bluish purple	white with yellow blotches	blackish
Filaments	1.5–2.0 mm, with short glandular hairs near base	ca. 1.2 mm, puberulent	ca. 1 mm, puberulent
Anthers	ovoid to ellipsoid, 3.0–3.5 mm long, apex brevirostrate	ellipsoid, ca. 3 mm, apex brevirostrate	broadly ellipsoid, ca. 3 mm, apex brevirostrate
Ovary	sparsely pubescent and with yellow glandular hairs	sparsely puberulent	minutely villous
Style	sparsely with yellow glandular hairs at base	sparsely puberulent near base	glabrous
Flowering	September to October	April to May	August to October

### 3. Didymocarpus brevipedunculatus

Taxon classificationPlantaeLamialesGesneriaceae

Y.H.Tan & Bin Yang sp. nov.

F57F6BA1D44C5A0C9D2646D55D509FA3

urn:lsid:ipni.org:names:60479353-2

Figures 5 , 6

#### Diagnosis.

*Didymocarpus brevipedunculatus* is similar to *D. purpureobracteatus* in bracts ovate to orbicular and calyx tubular, but it can be easily distinguished from the latter by its leaf base extremely obliquely cordate (vs. leaf base sometimes oblique, cuneate to cordate), inflorescence gracile, pendulous (vs. erect), inflorescence much shorter than leaf (vs. inflorescence much longer than leaf), peduncles (4.0–5.5 cm vs 4.0–10 cm long), flowers white with purplish to deep red longitudinal stripes (vs. purple to pinkish purple with darker stripes), and peduncles villous with eglandular, multicelluar hairs (vs. glabrous).

#### Type.

CHINA. Yunnan: Ximeng, Mengsuo, grows on rock surfaces along a seasonal waterfall or moist and shade places in evergreen forest, 22°38'04.83"N, 99°35'34.17"E, a.s.l. 1200 m, 8 September 2012, *Yun-Hong Tan 6930* (holotype: HITBC! Isotype: HITBC!).

**Figure 5.**
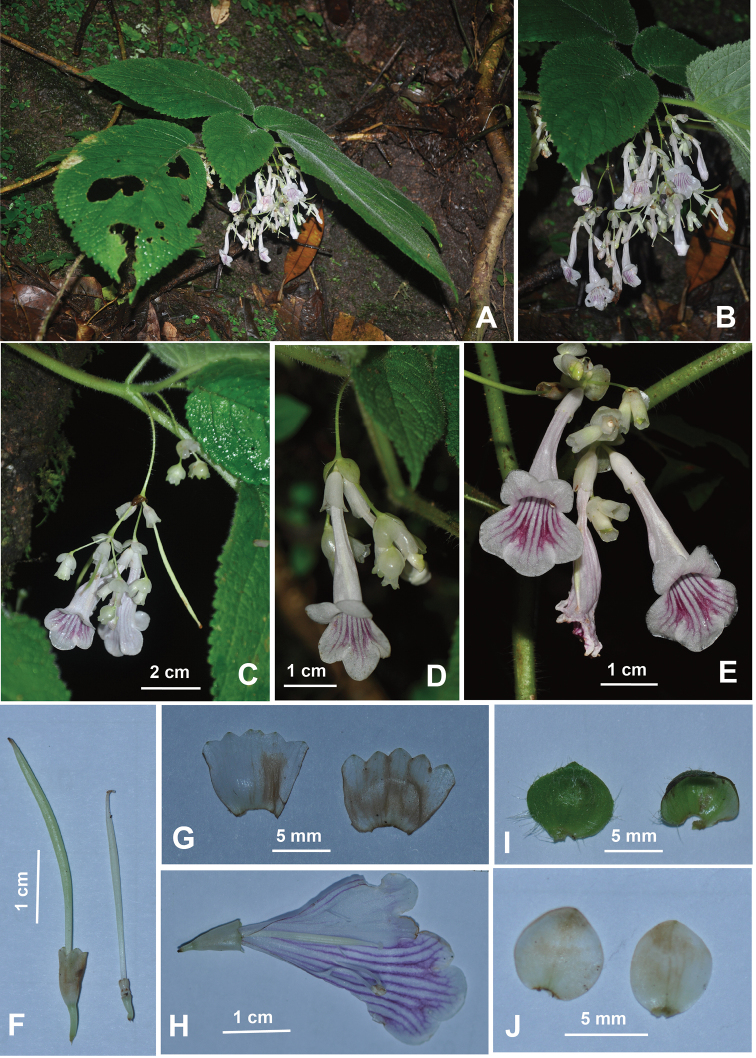
*Didymocarpus brevipedunculatus* Y.H.Tan & Bin Yang, sp. nov. **A, B** Habit **C, D** inflorescence **E** flowers **F** young capsules **G** dissected calyx **H** dissected corolla **I** bracts **J** bracteoles. Photographed by Y.H. Tan.

**Figure 6.**
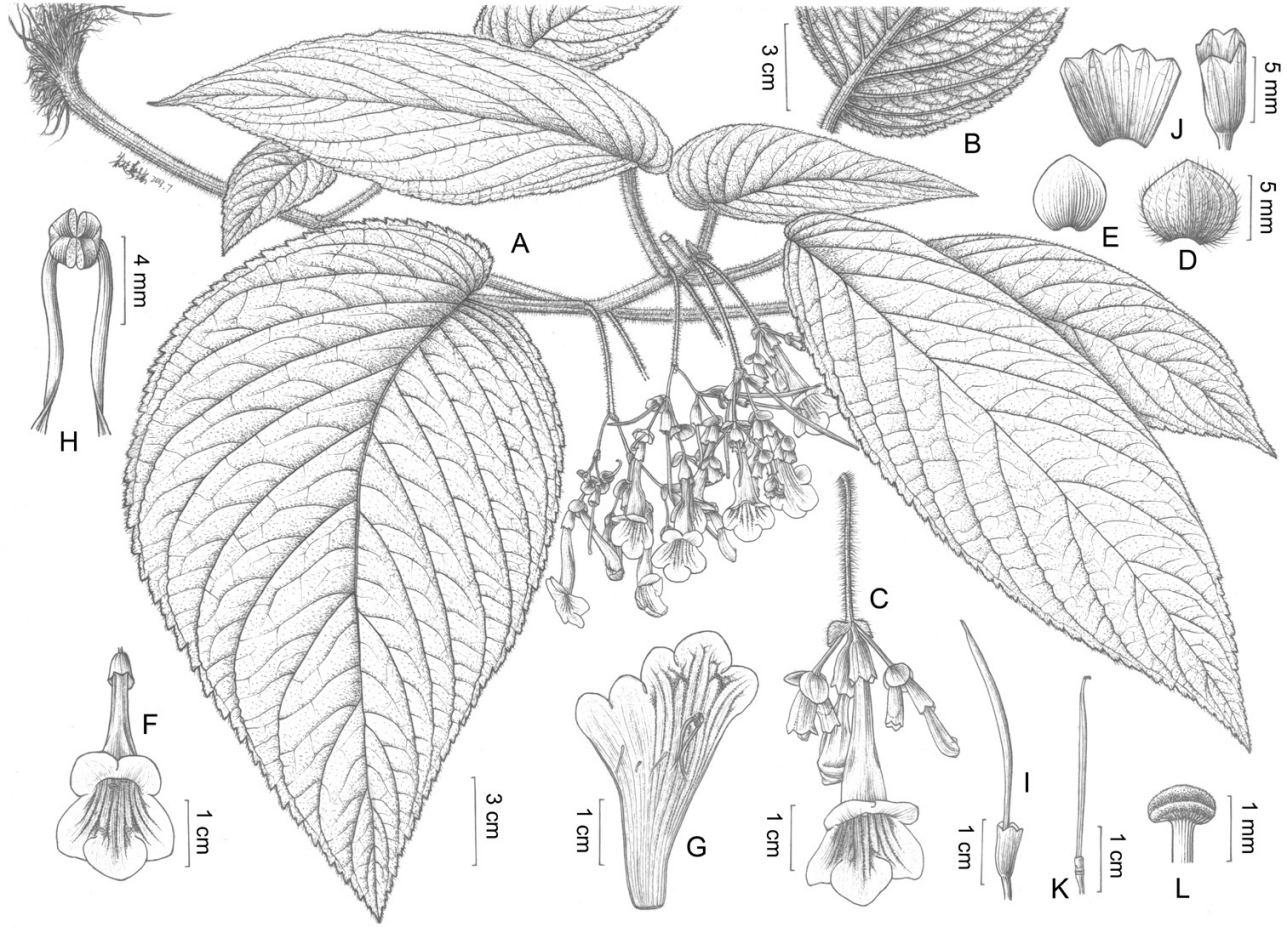
*Didymocarpus brevipedunculatus* Y.H.Tan, sp. nov. **A** Habit **B** leaf base in abaxial view **C** inflorescence **D** bract in abaxial view **E** bracteole **F** flower in front view **G** dissected corolla **H** stamens **I** young capsule **J** calyx **K** pistil **L** stigma. Drawn by Yun-xi Zhu.

#### Description.

Deciduous, perennial, epilithic herb, 30–40 cm tall, stem 4–6 mm in diameter. ***Dry season*** juvenile leaves distinct, blades symmetrically ovate, c. 1.5 × 1 cm, with much denser indumentum than when mature. ***Rainy season* stems** succulent, erect, green, densely and finely villous with multicellular eglandular hairs; pigment glands absent. **Leaves** 4–6 arranged in opposite, decussate, anisophyllous pairs; blades asymmetrically ovate, thin, papery when dry, upper surface dull dark green and drying medium brown, densely villous with eglandular, multicellular hairs, lower side pale light green and drying light brown, densely villous with eglandular, multicellular hairs along veins, 10–25 cm long, 6.5–15.5 cm wide, apex attenuate to acuminate, base extremely obliquely cordate, margins serrate, often irregularly so, or doubly serrate, midrib with 9–11 arching secondary veins on each side, distinct on both surfaces, finer venation reticulate; petioles 4.5–12.0 cm long, with indumentum as on the stems. **Inflorescence** solitary per axil, cymose, gracile, pendulous, 7–12 cm long, villous with eglandular, multicellular hairs, laxly cymose, axes succulent, light green to green; **Peduncles** 4.0–5.5 cm long, densely villous with eglandular, multicellular hairs; **Hypopodium** 1.0–2.0 cm long, glabrous or sparsely villous; **Pedicels** 3–5 mm long, glabrous or sparsely villous. **Bracts** paired; green to light green, sparsely villous with eglandular, multicellular hairs, orbicular to ovate, 5.5–6.0 mm long and wide. **Bracteoles** paired, whitish to light green, glabrous or sparsely villous, orbicular to ovate, 4.0–5.5 mm long and wide. **Flowers** numerous. **Calyx** campanulate, glabrous, often light green on both side, sometimes purplish outside; tube c. 6 mm long; lobes ovate, subequal to equal, 5(6) lobed, apices obtuse to rounded; 0.5–1.0 mm long. **Corolla** funnelform, 4.0–4.5 cm long, glabrous, white, inside with 9 purplish to deep red longitudinal stripes, 3 per lobe in the lower lip; tube 3.2–3.5 cm long, gradually widening from the base to the throat, 0.8–1.0 cm wide at base, 1.8–2.0 cm at throat; lobes ovate to suborbicular, broadly rounded; anterior (lower or abaxial) lip 3-lobed, 6–7 mm long, 7–8 mm wide apices rounded, posterior (upper or adaxial) lip 2-lobed 5–6 mm long, 7–8 mm wide, apices rounded. **Fertile stamens** 2, inserted at c. 2 cm above the base of the corolla; filaments 0.9–1.0 cm long, glabrous; anther locules oblong, c. 2 × 1 mm, tips and bases rounded, white-bearded, cream; **Staminodes** 3, inserted slightly below the stamens, lateral ones 5 mm long, the other one 3 mm long, glabrous. **Disc** ring-like, thickened, glabrous, margin entire or slightly lobed, 2–3 mm high, persistent in fruit. **Ovary** cylindric, slightly stipitate, glabrous, light green, c. 2.5–3.0 cm long, 1 mm wide; style continuous with the top of the ovary, c. 5 mm long, glabrous, whitish or light green; stigma discoid, concave medially, whitish, 1 mm diameter. **Capsules** cylindric, slightly stipitate, erect, straight, light green, when maturing light brown, 4.5–5 cm long and 2.5 mm wide. **Seeds**, numerous, elliptic, appendage absent, cell ornamentation straight, cell faces finely verrucate.

#### Etymology.

The new species is named after its axillary relatively short peduncles.

#### Vernacular.

Chinese mandarin: Duan Xu Chang Shuo Ju Tai (短序长蒴苣苔)

#### Phenology.

Flowering August-September and fruiting September-October.

#### Distribution and habitat.

The new species was found in south Yunnan, Ximeng and Cangyuan Counties. It grows on rock surfaces along a seasonal waterfall or in moist and shady places in evergreen forests, altitude 1000–1200 m.

#### Conservation status.

The localities of this new species, in Ximeng and Cangyuan, are both part of protected areas, and a total of more than one hundred individuals were found in the wild; a further inventory is needed to clarify the habitats and populations. At present, the species is therefore assigned a preliminary status of Endangered (EN D) according to the IUCN Red List Categories and Criteria (IUCN 2012).

#### Note.

A comparative list of diagnostic characters of the new species and *D. purpureobracteatus* is given in Table 3.

**Table 3.** Morphological comparison of *Didymocarpus brevipedunculatus* and its closely related species.

Characters	*D. brevipedunculatus*	*D. purpureobracteatus*
Shape of leaf Blade	asymmetrically ovate, base extremely obliquely cordate, apex attenuate to acuminate	symmetrically ovate to elliptic or obovate, base oblique, cuneate, to cordate, apex acute to acuminate
Leaf indumentum	upper surface densely villous with eglandular, multicellular hairs, lower side densely villous with eglandular, multicellular hairs along veins	adaxially sparsely appressed puberulent to nearly glabrous along veins, sparsely glandular
Petiole	4.5–12.0 cm long, densely villous with eglandular, multicellular hairs	0.3–11.0 cm long, puberulent, sparsely glandular
Bracts	orbicular to ovate, green to slightly green, sparely villous with eglandular, multicellular hairs	ovate to elliptic-ovate, sometimes connate at the base, galeate, covering calyx when flowering, glabrous
Calyx	6–7mm long, tubular campanulate, glabrous, lobe ovate to semiorbicular	10–12 mm long, tubular campanulate, glabrous, lobes semiorbicular
Inflorescence	gracile, pendulous, much shorter than leave	erect, much longer than leave
Peduncle	4.0–5.5 cm long	4.0–10.0 cm long
Corolla	white, inside with purplish to deep red longitudinal stripes	purple to pinkish purple with darker stripes, glabrous, corolla tube funnelform
Filaments	0.9–1 cm long, glabrous	ca. 1 cm, glabrous
Staminode	three, 1.0–3.0 mm long	two, 1.5–3.0 mm long

### 4. Henckelia xinpingensis

Taxon classificationPlantaeLamialesGesneriaceae

Y.H.Tan & Bin Yang sp. nov.

3F81FCC8DFD65533AFDEC218FFC8DEB1

urn:lsid:ipni.org:names:60479354-2

**Figure 7.**
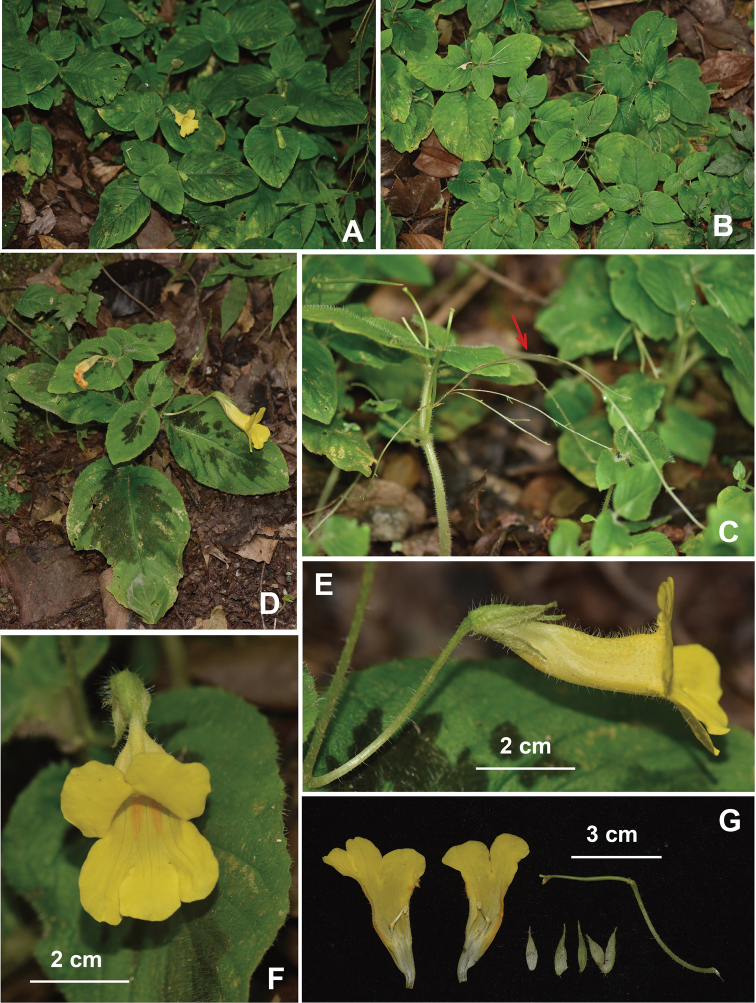
*Henckelia xinpingensis* Y.H.Tan & Bin Yang, sp. nov. **A, B, D** Habit **C** habit (showing stolons) **E** flower in side view **F** flower in front view **G** dissected flower. Photographed by B.Yang.

**Figure 8.**
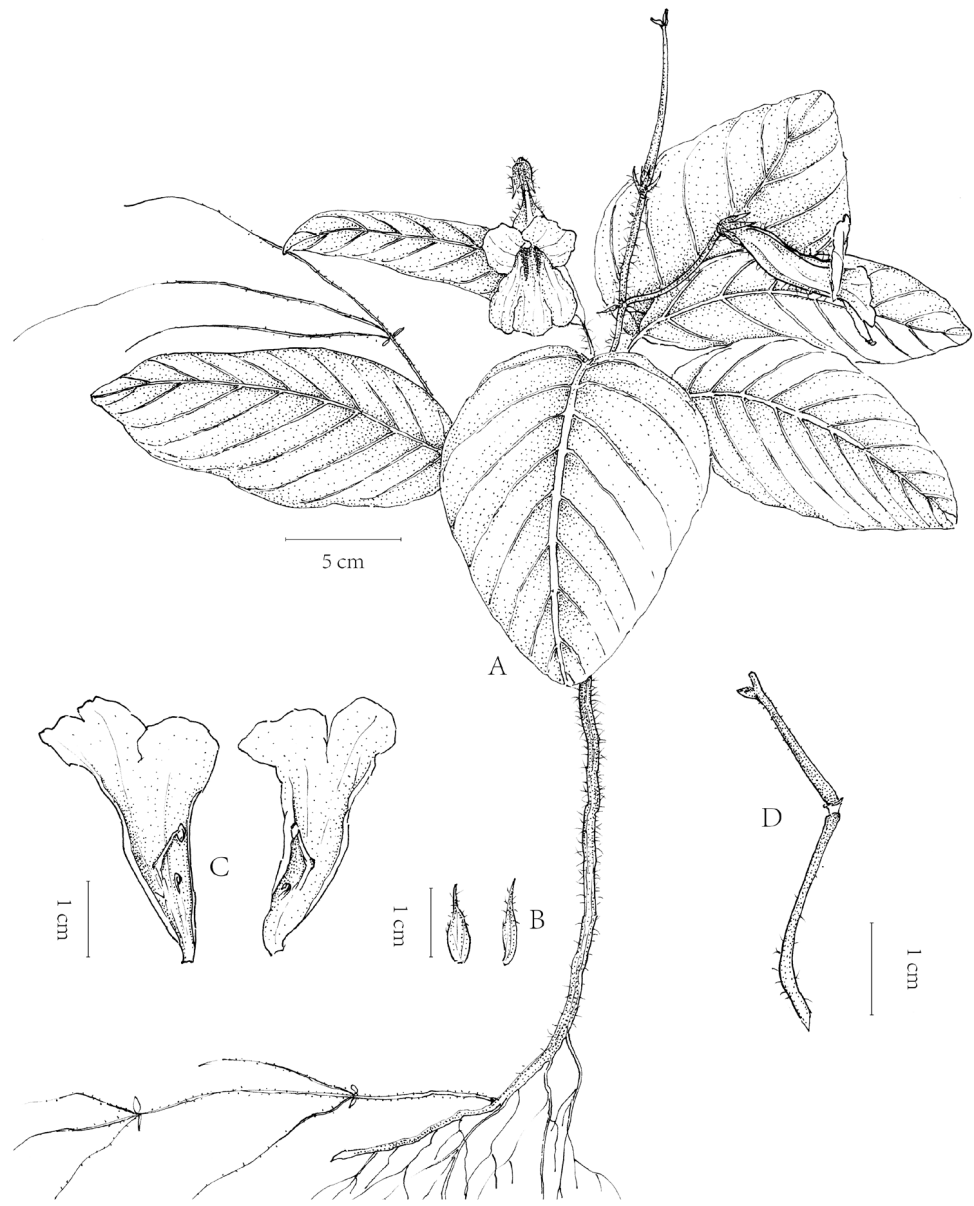
*Henckelia xinpingensis* Y.H.Tan & Bin Yang, sp. nov. **A** Habit **B** calyx lobes **C** corolla (Dissected) **D** pedicel with pistil. Drawn by Zheng-meng Yang.

**Figure 9.**
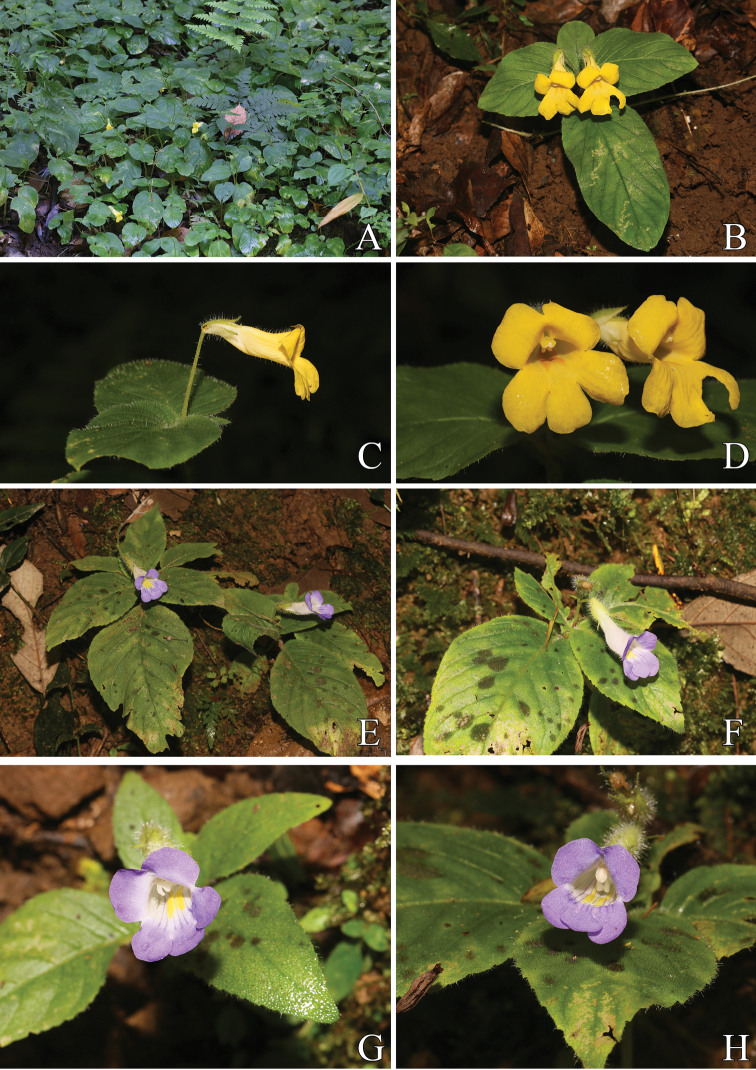
*Henckelia xinpingensis* Y.H.Tan & Bin Yang, sp. nov. (**A–D**) **A, B** Habit **C** flower (side view) **D** flower (front view); *Henckelia pumila* (D. Don) A. Dietr. (**E–H**) **E, F** habit **G, H** flower (front view). Photographed by H.B. Ding.

Figures 7 , 8 , 9 (A1–A4)

#### Diagnosis.

*Henckelia xinpingensis* is similar to *H. pumila* in having elliptic leaf blades sometimes with purple spots abaxially, appearing brown-green adaxially, and funnel form corolla, but differs in having intensive yellow (vs. white to purple) corollas, stigma undivided or slightly 2-lobed (vs. conspicuous 2-lobed), calyx from base to below the middle (vs. 5-lobed from below to above middle); leaf blade symmetrical, base rounded to cordate (vs. asymmetrical, base oblique) and producing slender stolons.

#### Type.

CHINA. Yunnan Province: Xinping county, Yubaiding, 24°09.32'N, 102°07.71'E, a.s.l. 1500 m, 17 Aug. 2018, Y.H. Tan, B. Yang, H.B. Ding & X.D. Zeng *Y0130* (holotype: HITBC!).

#### Description.

Annual herbs, usually producing slender stolons from stem base, leaf axils or occasionally bract axils, stolons 10–25 cm, pubescent. **Stems** erect, 5–25 cm, pubescent to sparsely pilose. **Leaves** 4–6, opposite, widely spaced nodes; petiole 0.5–3.5 cm; blade symmetrical, ovate-elliptic to elliptic, 2–15 × 1.2–8.0 cm, herbaceous, puberulous to sparsely pilose, eglandular, abaxially sometimes with purple spots, adaxially appearing brown-green, base rounded to cordate, margin repand to entire, apex acute or obtuse; lateral veins 5–9 on each side of midrib, conspicuous. **Cymes** 1–4-flowered; **Peduncle** 0.5–3.5 cm, sparsely pilose; **Bracts** 2, free, linear to lanceolate, 3–6 mm long. **Pedicel** 2.5–5.0 cm, sparsely pilose. **Calyx** 1.2–1.7 cm, narrowly bell-shaped, divided into 5 lobes from base to below the middle; tube 3.5–4 mm; lobes subequal, lanceolate, 12–14 × 2–3 mm, outside sparsely pilose, inside glabrous, margin entire, apex subulate-attenuate. **Corolla** intensive yellow with two yellow-orange stripes on the abaxial lip, 3.7–4.2 cm long, outside sparsely glandular pilose, inside glabrous; tube narrowly funnelform, 3.4–3.8 × 0.9–1.2 cm; adaxial lip 1.9–2.3 × 0.8–1.0 cm, 2-lobed, abaxial lip 2.5–3.0 × 0.9–1.2 cm, 3-lobed, all lobes semi-orbicular, with rounded apex. **Stamens** 2, 1.3–1.5 cm long, adnate to the corolla tube below middle; filaments 1.1–1.3 cm long, sparsely puberulent to glabrous, bending in the middle, with knee; anthers fused by entire adaxial surfaces, ca. 3.5 mm, glabrous, dediscence; **Staminodes** 3, 2.5–6.0 mm. **Pistil** 2.5–2.8 cm, sparsely puberulent to puberulous, with short glandular hairs near apex; ovary 2.2–2.5cm; style 3–6 mm long, sparsely glandular puberulent. **Stigma** flabellate, 2–3 mm, undivided or slightly 2-lobed. **Capsule** sub-erect, 5–10 cm, loculicidal dehiscence .

#### Etymology.

The new species is named after its type locality Xinping County.

#### Vernacular name.

Chinese mandarin: Xin Ping Chun Zhu Ju Tai (新平唇柱苣苔).

#### Phenology.

Flowering in August and fruiting from August to September.

#### Distribution and habitat.

This species is only known from Xinping county, but is relatively common there growing in moist areas near stream sides and roadsides under the subtropical broad leaf forests.

#### Additional specimens examined

**(paratypes).** CHINA. Yunnan Province: Xinping, Dapingzhang, 102°04.435'E, 24°04.672'N, a.s.l. 580 m, 16 Aug. 2018, Y.H. Tan, B. Yang *Y0115* (HITBC!); Ibid., 16 Aug. 2018, Y.H. Tan & B. Yang *Y0118* (HITBC!).

#### Conservation status.

According to our field observations, more than ten populations have been observed around an area of 20 hectares and each population of the new species has more than 100 individuals. The species is therefore assigned a preliminary status of Least Concern (LC) according to the IUCN Red List Categories and Criteria (IUCN 2012).

#### Note.

*Henckelia xinpingensis* has elliptic leaf blades with a pilose indumentum similar to *H. pumila*. A comparative list of diagnostic characters of the new species and *H. pumila* is given in Table 4.

**Table 4.** Morphological comparison of *Henckelia xinpingensis* and its closely related species.

Characters	*H. xinpingensis*	*H. pumila*
Habit	producing slender stolons	not stolons
Leaf blade	symmetrical, base rounded to cordate, ovate-elliptic to elliptic, 2–15 × 1.2–8.0 cm	asymmetrical, base oblique, lanceolate to ovate or elliptic, 2–17 × 1.2–5.5(–8.0) cm
Leaf margin	repand to entire	denticulate to serrulate
Cymes	1–4-flowered	(1 or) 2–7 -flowered
Peduncle	0.5–3.5 cm	2.8–10.0 cm
Bracts	2, free, linear to lanceolate, 3–6 × 1–3 mm	2, free, ovate to lanceolate or obovate, 5–18 × 1–4 mm
Pedicel	2.5–5.0 cm	0.3–2.0 cm
Calyx	1.2–1.7 cm, 5-lobed nearly to base or below the middle; tube 0.5–4.0 mm	0.9–1.8 cm, 5-lobed to middle or slightly below; tube 4–10 mm
Calyx lobes	subequal, lanceolate, 12–14 × 2–3 mm, apex subulate-attenuate	slightly unequal, narrowly triangular to ovate, 4–10 × ca. 2 mm, apex subulate-acuminate, hornlike, spreading
Corolla	intensive yellow, outside glandular pilose	white to purple, outside puberulent to pilose,
Pistil	2.5–2.8 cm long, with short glandular hairs near apex	2.5–3.8 cm long, glabrous to puberulent
Stigma	labellate, 2–3 mm, undivided or slightly 2-lobed	flabellate, ca. 3 mm, conspicuous 2-lobed
Capsule	5–10 cm	6–12 cm

## Supplementary Material

XML Treatment for Petrocosmea rhombifolia

XML Treatment for Petrocosmea tsaii

XML Treatment for Didymocarpus brevipedunculatus

XML Treatment for Henckelia xinpingensis

## References

[B1] Burtt BL (1998a) A new species of *Petrocosmea*. The Gloxinian 1: 14–15.

[B2] Burtt BL (1998b) Taxonomic history of *Didymocarpus* and *Henckelia* (Gesneriaceae). Beitrge zur Biologie der Pflanzen 70: 365–375.

[B3] Burtt BL (1999) Old World Gesneriaceae. VI. Six miscellaneous notes. Edinburgh Journal of Botany 56(3): 371–379. 10.1017/S0960428600001335

[B4] Burtt BL (2001) Flora of Thailand: Annotated checklist of Gesneriaceae. Thai Forest Bulletin (Botany) 29: 81–109.

[B5] Cai L Cai J Shui YM (2016) *Didymocarpus anningensis* (Gesneriaceae), a new species from Yunnan, China. Phytotaxa 255(3): 292–296. 10.11646/phytotaxa.255.3.12

[B6] Craib WG (1918) Contributions to the Flora of Siam. Additamentum X. Bulletin of Miscellaneous Information (Royal Botanic Gardens, Kew) 10: 362–371. 10.2307/4111886

[B7] Dietrich A (1831) Caroli A Linne Species Plantarum exhibentes Plantas Rite Cognitas ad Genera Relatas, Editio sexta 1. Impensis G.C. Nauck, Berolini, 735 pp.

[B8] Gou GQ Wang XY Xiong YX (2010) *Petrocosmea xanthomaculata* G Q. Gou et A Y. Wang, a new species of the genus *Petrocosmea* Oliv. Bulletin of Botanical Research 30: 394–396.

[B9] Han MQ Lü TF Liu Y (2017) *Petrocosmea magnifica* (Gesneriaceae): A new species from limestone caves in Yunnan, China. Phytotaxa 319(3): 283–288. 10.11646/phytotaxa.319.3.8

[B10] Han MQ Lü TF Liu Y (2018a) *Petrocosmea viridis* sp. nov. of *Petrocosmea* (Gesneriaceae) from Guizhou, China and a supplementary and revised description of *P. minor* Nordic Journal of Botany 2018(3): e01566. 10.1111/njb.01566

[B11] Han MQ Yuan Q Lü TF Jiang H Liu Y (2018b) *Petrocosmea chrysotricha* sp. nov. (*Petrocosmea*, Gesneriaceae), a species previously mistaken for *P. begoniifolia* on marlstone cliffs in Yunnan, China. Nordic Journal of Botany 2018(4): e01664. 10.1111/njb.01664

[B12] Hilliard OM (2001) Gesneriaceae. In: Grierson AJC Long DG Springate LS (Eds) Flora of Bhutan.Vol 2. Part 3. Royal Botanic Garden, Edinburgh, 1296–1330. 10.1017/S0960428600000974

[B13] Hong X Li ZL Maciejewski S Wen F Do TV (2018) *Didymocarpus puhoatensis* (Gesneriaceae), a new species from Vietnam. In: Jin XH Shui YM Tan YH Kang M (Eds) Plant diversity in Southeast Asia.PhytoKeys 94: 87–93. 10.3897/phytokeys.94.21650 PMC5799735 29416423

[B14] IUCN (2012) IUCN Red List Categories and Criteria: Version 3.1. IUCN; Gland, Switzerland and Cambridge, UK: 32.

[B15] Subcommittee IUCN Standards and Petitions (2016) Guidelines for using the IUCN Red List categories and criteria. Version 12. IUCN; 101. http://www.iucnredlist.org/documents/RedListGuidelines.pdf [accessed 29 March 2016]

[B16] Joe A Hareesh VS Prashob P Sabu M (2016) *Didymocarpus moellerii* (Gesneriaceae): A new species from northeastern India. Phytotaxa 266(1): 57–60. 10.11646/phytotaxa.266.1.10

[B17] Li HW (1983) Notulae de Gesneriaceis yunnanensibus. Bulletin of Botanical Research 3: 1–55.

[B18] Li JM Li SL (2014) Didymocarpus heucherifolius var. yinzhengii (Gesneriaceae), a new taxon from Hunan, China. Phytotaxa 156(3): 187–190. 10.11646/phytotaxa.156.3.10

[B19] Li JM Wang FS (2015) *Didymocarpus tonghaiensis* sp. nov. (Gesneriaceae) from Yunnan, China. Nordic Journal of Botany 33(1): 68–70. 10.1111/njb.00465

[B20] Li ZY Wang YZ (2004) Plants of Gesneriaceae in China. Henan Science and Technology Publishing House, Zhengzhou, 721 pp.

[B21] Middleton DJ Triboun P (2010) Two new species of *Petrocosmea* (Gesneriaceae) from Thailand. Thai Forest Bulletin (Botany) 38: 42–47.

[B22] Middleton DJ Weber A Yao TL Sontag S Möller M (2013) The current status of the species hitherto assigned to *Henckelia* (Gesneriaceae). Edinburgh Journal of Botany 70(3): 385–404. 10.1017/S0960428613000127

[B23] Middleton DJ Khew GS Poopath M Möller M Puglisi C (2018) *Rachunia cymbiformis*, a new genus and species of Gesneriaceae from Thailand. Nordic Journal of Botany 2018(11): e01992. 10.1111/njb.01992

[B24] Möller M Wei YG Wen F Clark JL Weber A (2016a) You win some you lose some: Updated generic delineations and classification of Gesneriaceae-implications for the family in China. Guihaia 36: 44–60.

[B25] Möller M Nishii K Atkins HJ Kong HH Kang M Wei YG Wen F Hong X Middleton DJ (2016b) An expansion of the genus *Deinostigma* (Gesneriaceae). Gardens’ Bulletin (Singapore) 68(1): 145–172. 10.3850/S2382581216000119

[B26] Nangngam P Maxwell JF (2013) *Didymocarpus* (Gesneriaceae) in Thailand. Gardens’ Bulletin (Singapore) 65: 185–225.

[B27] Nangngam P Middleton DJ (2014) Five new species of *Didymocarpus* (Gesneriaceae) from Thailand. Thai Forest Bulletin (Botany) 42: 35–42.

[B28] Oliver D (1887) *Petrocosmea sinensis* Oliv. In: Hooker WD (Ed.) Icones Plantarum 18. Longman, London, pl. 1716.

[B29] Phuong VX Dang QV Xuyen DT (2014) Genus *Didymocarpus* Wall. and a new record of species *Didymocarpus purpureobracteatus* Smith for the flora of Vietnam from Xuan Lien natural reserve, Thanh Hoa province. Tap Chi Sinh Hoc 36(1): 45–49. 10.15625/0866-7160/v36n1.4516

[B30] Qiu ZJ Li CQ Wang YZ (2015a) *Petrocosmea glabristoma* (Gesneriaceae), a new species from Yunnan, China. Plant Diversity and Resources 37: 551–556.

[B31] Qiu ZJ Liu ZY (2015) Plants of *Petrocosmea* in China. Science Press, Beijing, 382 pp.

[B32] Qiu ZJ Lu YX Li CQ Dong Y Smith JF Wang YZ (2015b) Origin and evolution of *Petrocosmea* (Gesneriaceae) inferred from both DNA sequence and novel findings in morphology with a test of morphology-based hypotheses. BMC Plant Biology 15(1): 167 10.1186/s12870-015-0540-3 26135135 PMC4489212

[B33] Qiu ZJ Wang XL Liu ZY Yang JF Zhang SZ (2012) Cytological and phylogenetic study of *Petrocosmea hexiensis* (Gesneriaceae), a new species from Chongqing, China. Phytotaxa 74(1): 30–38. 10.11646/phytotaxa.74.1.2

[B34] Qiu ZJ Yuan ZL Li ZY Wang YZ (2011) Confirmation of a natural hybrid species in *Petrocosmea* (Gesneriaceae) based on molecular and morphological evidence. Journal of Systematics and Evolution 49(5): 449–463. 10.1111/j.1759-6831.2011.00151.x

[B35] Ranasinghe S Milne R Jayasekara R Rubasinghe S Möller M (2016) *Henckelia wijesundarae* (Gesneriaceae), a new endemic species from Sri Lanka, and lectotypification of *Chirita walkerae* and C. walkerae var. parviflora. Willdenowia 46(2): 213–224. 10.3372/wi.46.46202

[B36] Shaw J (2011) A new species of *Petrocosmea*. Plantsman (London, England) 10: 177–179.

[B37] Sirimongkol S Parnel JAN Hodkinson TR Middleton DJ Puglisi C (2019) Five new species of *Henckelia* (Gesneriaceae) from Myanmar and Thailand. Thai Forest Bulletin (Botany) 47(1): 38–54. 10.20531/tfb.2019.47.1.08

[B38] Smith WW (1912) New Burmo-Chinese species of *Didymocarpus*. Notes from the Royal Botanic Garden Edinburgh 5: 149–156.

[B39] Sprengel CPJ (1817) Anleitung zur Kenntniss der Gewächse (2^nd^ edn). Part 2(1), 502 pp.

[B40] Wallich N (1819) Notice of the progress of botanical science in Bengal, being the substance of a letter from Dr. Wallich to Francis Hamilton. Edinburgh Philosophical Journal 1: 376–378.

[B41] Wang HC Zhang LB He ZR (2013) *Petrocosmea melanophthalma*, a new species in section Deianthera (Gesneriaceae) from Yunnan, China. Novon 22(4): 486–490. 10.3417/2011035

[B42] Wang WT (1985) The second revision of the genus *Petrocosmea* (Gesneriaceae). Acta Botanica Yunnanica 7: 49–68.

[B43] Wang WT Pan KY Li ZY (1990) Gesneriaceae. In: Wang WT (Ed.) Flora Reipublicae Popularis Sinicae Vol.69. Science Press, Beijing, 190–203.

[B44] Wang WT Pan KY Li ZY Weitzman AL Skog LE (1998) Gesneriaceae. In: Wu ZH Raven PH (Eds) Flora of China.Vol.18. Science Press, Beijing, and Missouri Botanical Garden Press, St. Louis, 244–401.

[B45] Weber A Burtt BL (1998) Remodeling of *Didymocarpus* and associated genera (Gesneriaceae). Beiträge zur Biologie der Pflanzen 70: 293–363.

[B46] Weber A Burtt BL Vitek E (2000) Materials for a revision of *Didymocarpus* (Gesneriaceae). Annalen des Naturhistorischen Museums in Wien 102B: 441–475.

[B47] Weber A Middleton DJ Forrest A Kiew R Lim CL Rafidah AR Sontag S Triboun P Wei YG Yao TL Möller M (2011) Molecular systematics and remodelling of *Chirita* and associated genera (Gesneriaceae). Taxon 60(3): 767–790. 10.1002/tax.603012

[B48] Weber A Clark JL Möller M (2013) A new formal classification of Gesneriaceae. Selbyana 31(2): 68–94.

[B49] Wen F Qiu YL Huang J Zhao B Wei YG (2013) *Didymocarpus dissectus* sp. nov. (Gesneriaceae) from Fujian, eastern China. Nordic Journal of Botany 31(3): 316–320. 10.1111/j.1756-1051.2012.00057.x

[B50] Wei YG Wen F (2009) *Petrocosmea xingyiensis* (Gesneriaceae), a new species from Guizhou, China. Novon 19(2): 261–262. 10.3417/2007090

[B51] Xu WB Pan B Liu Y (2011) *Petrocosmea huanjiangensis*, a new species of Gesneriaceae from limestone areas in Guangxi, China. Novon 21(3): 385–387. 10.3417/2009101

[B52] Xu WB Guo J Pan B Zhang Q Liu Y (2017) Diversity and distribution of Gesneriaceae in China. Guihaia 37: 1219–1226.

[B53] Zhang Q Pan B Meng T Li GF Xu WB Li ZM (2013) *Petrocosmea funingensis* (Gesneriaceae): A new species from southeastern Yunnan. China. Phytotaxa 77(1): 5–8. 10.11646/phytotaxa.77.1.2

[B54] Zhao HT Shui YM (2010) *Petrocosmea shilinensis*, a new species of Gesneriaceae from Yunnan, China. Acta Botanica Yunnanica 32: 328–330.

